# Novel Treatment of Warts

**Published:** 1886-10-20

**Authors:** 


**Novel Treatment of Warts.**—"Thuya occidentalis" (arbor vitæ) is credited with the remarkable property of causing the disappearance, in a very short time, of all kinds of vegetations and warty growths, by its internal administration. Many successful cases are reported by French physicians; in fact, it is said to seldom or never fail. It is given in the form of tincture, from ʒ ss to ʒ j, two or three times a day. Its action is regarded as quite phenomenal, in this direction. Among others, Dr. Constantin Paul reports the cure, within fourteen days, of non-syphilitic warts which covered the gentalia of a woman. It is said to be equally serviceable in the case of condylomata and other similar growth.—*Medical Wortd.*

